# Assessment of the latest NGS enrichment capture methods in clinical context

**DOI:** 10.1038/srep20948

**Published:** 2016-02-11

**Authors:** Gema García-García, David Baux, Valérie Faugère, Mélody Moclyn, Michel Koenig, Mireille Claustres, Anne-Françoise Roux

**Affiliations:** 1 Laboratoire de génétique de maladies rares, EA 7402, Université de Montpellier, Montpellier, France; 2 Laboratoire de génétique moléculaire, CHRU Montpelier, Montpellier, France

## Abstract

Enrichment capture methods for NGS are widely used, however, they evolve rapidly and it is necessary to periodically measure their strengths and weaknesses before transfer to diagnostic services. We assessed two recently released custom DNA solution-capture enrichment methods for NGS, namely Illumina NRCCE and Agilent SureSelect^QXT^, against a reference method NimbleGen SeqCap EZ Choice on a similar gene panel, sharing 678 kb and 110 genes. Two Illumina MiSeq runs of 12 samples each have been performed, for each of the three methods, using the same 24 patients (affected with sensorineural disorders). Technical outcomes have been computed and compared, including depth and evenness of coverage, enrichment in targeted regions, performance in GC-rich regions and ability to generate consistent variant datasets. While we show that the three methods resulted in suitable datasets for standard DNA variant discovery, we describe significant differences between the results for the above parameters. NimbleGen offered the best depth of coverage and evenness, while NRCCE showed the highest on target levels but high duplicate rates. SureSelect^QXT^ showed an overall quality close to that of NimbleGen. The new methods exhibit reduced preparation time but behave differently. These findings will guide laboratories in their choice of library enrichment approach.

Custom targeted gene sequencing may become the standard in clinical practice for genetically heterogeneous diseases, as it brings the high capacity of 2^nd^ generation sequencing (Next Generation Sequencing, NGS) to molecular diagnostic laboratories. The approach offers several advantages in such applications compared with whole exome/genome sequencing (WES, WGS). Despite the decreasing costs of NGS technologies, WGS remains expensive and bioinformatics analysis a bottleneck to implement it in molecular diagnostic laboratories^1^. WES is a more accessible strategy suitable for molecular diagnosis of heterogeneous diseases and for discovering novel disease related genes^2^,^3^. Nevertheless, custom targeted sequencing has several advantages in such applications compared with WES and WGS. These are either technical^4^,^5^,^6^,^7^, financial (more samples per run) or even ethical by reducing the risks of identifying incidental findings^8^. However, the main advantage of custom targeted sequencing remains the possibility to personalize the design (i.e., inclusion of complete gene sequence or specific intronic sequences).

Sequencing of custom gene panels can be performed using libraries enriched with sequences of interest (generated with solid or liquid hybridization methods) or multiplexed PCR-based methods. Recently, Samorodnitsky *et al.*^9^ assessed the efficiency of two hybridization (capture) and two amplicon-based methods in WES context and showed that capture systems still perform better in terms of uniformity of coverage, sequencing complexity and ability to call true SNVs than amplicon-based methods.

Many studies compared the most widely used capture-based methods, mainly in WES approach, namely SureSelect (Agilent,) TruSeq Capture (Illumina) and SeqCap EZ (Roche NimbleGen). Most studies showed minor differences^10^,^11^,^12^ or reported a higher coverage and efficiency with NimbleGen^13^,^14^,^15^. Conversely, a recent study^16^ compared the latest exome version of these three companies and the authors concluded in a global better performance and robustness of SureSelect, highlighting the importance of these updated comparisons with new protocols.

Some studies focused on custom capture^17^,^18^,^19^, but the field is evolving rapidly, as new methods that require less DNA input and using enzymatic fragmentation with a reduced preparation time have since been released.

We present here the assessment of two of these recent methods, Agilent SureSelect^QXT^ (named SureSelect^QXT^ herein) and Illumina Nextera Rapid Custom Capture Enrichment (NRCCE), using our custom gene panel designed to test for genetic defects in patients presenting with Non Syndromic Hearing Loss (NSHL), Usher syndrome (USH, a recessive sensorineural disorder defined by the association of congenital HL and retinitis pigmentosa later on) and Autosomal Recessive Retinitis Pigmentosa (ARRP). Using the conventional NimbleGen SeqCap EZ Choice (NimbleGen) method as reference, we applied the three protocols to a group of 24 patients and systematically compared critical parameters susceptible to have an impact on the quality of the results. These parameters were chosen following NGS practice guidelines^20^,^21^,^22^. While we report differences in several aspects, all three methods resulted in datasets suitable for proper DNA variants detection.

## Results

### Raw data

**Figure 1:**
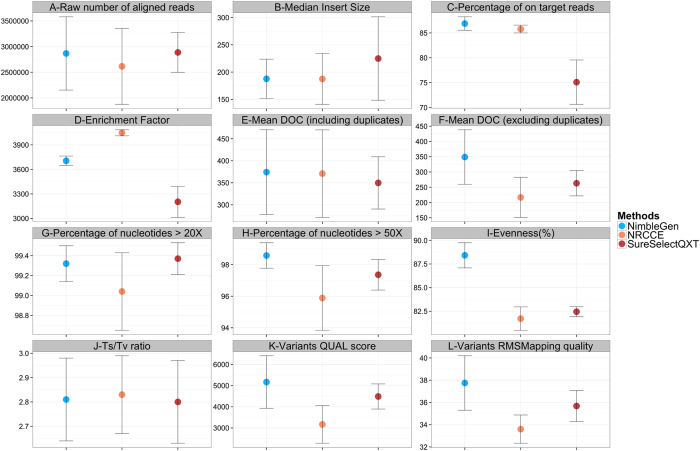
Main parameters considered for quality assessment of the three NGS protocols.

About 1000 k/mm2 clusters (963–1090 k/mm2) were generated per run, producing approximately 5 Gb (4.80–5.20) of bases sequenced and 2.5–3 millions of aligned reads (Fig. 1A, Supplementary Table S3). The six runs were of high quality with a mean of more than 89% of sequenced bases with a Phred Q score > = 30 (Supplementary Table S3).

The mean median size and distribution of sequenced fragments was significantly higher for SureSelect^QXT^ runs compared to NRCCE and NimbleGen (p-values < 0.01 in all cases, Fig. 1B, Supplementary Table S3).

The proportion of PCR duplicate reads represented one of the major discrepancy between the different methods. While only 7.7% of the reads were identified as PCR duplicates with NimbleGen, this percentage was clearly higher for SureSelect^QXT^ (18.5%) and NRCCE (41.2%, see also Supplementary Table S3). We investigated the localisation of the duplicate reads per gene (Supplementary Fig. S1), and observed an even distribution for the three methods.

### On target percentage and Enrichment factor

The proportion of reads falling into targeted regions was similar for NRCCE and NimbleGen (85% and 86%, respectively). This percentage was lower for SureSelect^QXT^, with 75% of reads mapped in targeted regions (Fig. 1C, Supplementary Table S3).

The difference in target size between the 3 designs might have an impact on the on target percentage. To avoid such biases, we computed the Enrichment Factor (EF)^18^. High EF values denote high capacity of the method to capture regions of interest. The EF was correlated with the on target percentage described above, as NRCCE presented the highest EF (4,048), close to NimbleGen (3,705). SureSelect^QXT^ had a slightly lower EF (3,203) (Fig. 1D and Supplementary Table S3).

### Coverage and Evenness score

We first estimated the general raw depth and all three methods appeared to have a similar mean DOC of about 370X (Fig. 1E, Supplementary Table S3). However, when duplicates were removed the results changed drastically. The most affected method was NRCCE, with a mean DOC decreasing from 371X to 217X (minus 154X). In the case of SureSelect^QXT^ the difference was −87X, while it was only of −25X for NimbleGen (Fig. 1F, Supplementary Table S3).

For the 3 methods, 99% of targeted nucleotides were covered at more than 20X (Fig. 1G, Supplementary Table S3). However, when we consider the same percentage at more than 50X, the results were slightly lower for NRCCE, which also showed a highest dispersion (Fig. 1H, Supplementary Table S3).

**Figure 2:**
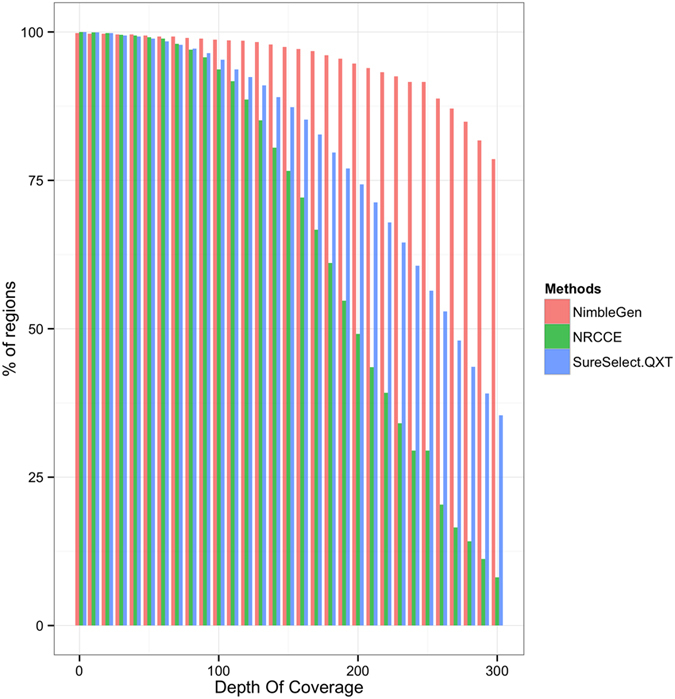
Comparison of coverage regions distribution for the three NGS protocols. The figure represents the percentage of bases covered at fixed depths.

Figure 2 shows the percentage of regions covered at different depths. In all cases only a few regions were poorly covered (from 7 to 14 regions covered <40X) but regions with low coverage were not always the same in the 3 techniques. More than 90% of the regions showed a DOC > 100X for all methods. However, strong differences appeared in higher depths: while NimbleGen still achieves more than 80% of regions with a DOC > 280X, SureSelect^QXT^ falls to less than 40% and NRCCE to less than 15% for an equivalent depth.

These results were confirmed when examining the uniformity of the coverage by calculating the evenness score (ES)^23^. ES is here presented as a percentage, a value of 100% representing a perfect uniformity. If the three methods gave results above 80%, unsurprisingly NimbleGen presented the highest ES (88.42%) (Fig. 1I, Supplementary Table S3). The uniformity of the coverage distribution was also examined in Supplementary Fig. S2, which shows the normalized DOC per region and highlights a clearly weaker dispersion for NimbleGen.

### GC content

**Figure 3:**
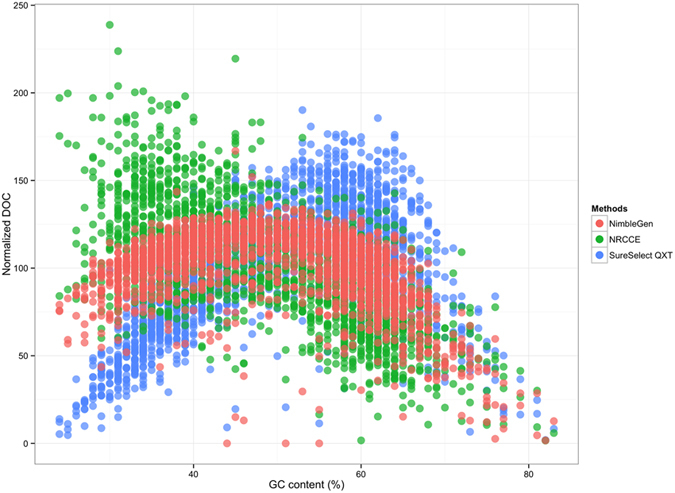
Normalized depth of coverage (DOC) in different GC content contexts. Normalization was obtained by dividing each region DOC by the average DOC for the considered method.

Performance in variable GC content was assessed using a density plot showing the distribution of the normalized DOC with increasing GC content (Fig. 3). As expected, for all protocols the DOC dropped out along with the GC content increase (>65–70%).

While SureSelect^QXT^ presents higher DOC in high GC content conditions, NRCCE is the most performing one in low GC-content context (30–40%).

### Variant calling

**Figure 4:**
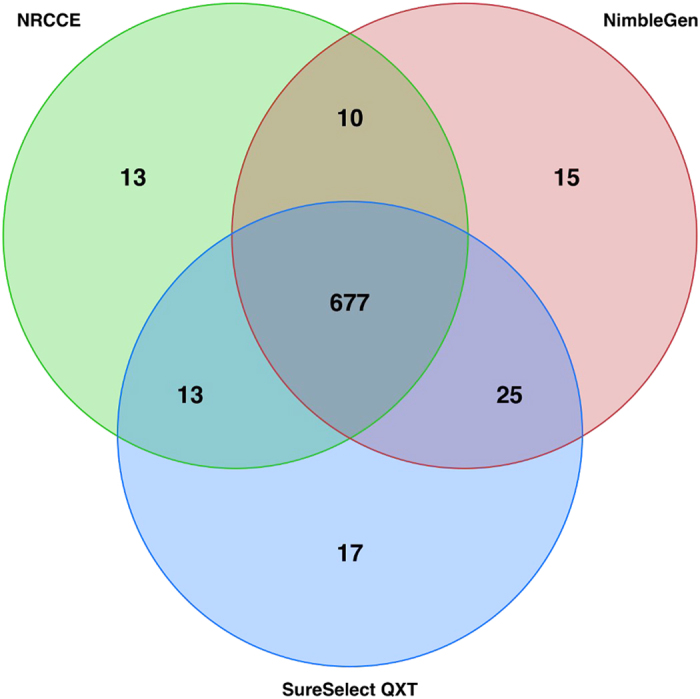
Venn diagram of the number of variants identified with the three NGS protocols in the same genomic regions.

The number of variants reported in each method was very similar (Supplementary Table S3) and a mean of 677 variants were common for each patient (Fig. 4). About 10–25 variants were only found by one or two methods.

All variants previously identified by Sanger sequencing (n = 113) and all putative pathogenic variants identified during the analysis (n = 20, including 4 at homozygous state), with the exception of one, were identified by the 3 methods (Supplementary Table S4). The possible pathogenic variants were of several types and include substitutions, single or multiple bp deletions and a homozygous deletion of a complete exon. The latter were confirmed either by Sanger sequencing or by Multiplex Ligation-dependent Probe Amplification (MLPA) for the complete exon deletion.

One single in frame deletion of 6 bp in the *MYO7A* gene was not reported in the VCF file generated with NRCCE while it was clearly identified with the 2 other methods. Visual inspection of the BAM files using IGV showed a reduced amount of reads carrying the deletion in NRCCE compared with the 2 others (around 20% of reads against more than 35%), but that could not explain its complete absence in the VCF file. Results were similar when the sample was reanalysed with an updated version of MSR (v2.5.1). We finally reanalysed with a custom pipeline, and the deletion was identified with an allele balance of 18.9%.

We assessed the allele balance associated with the variant calls (filter ‘PASS’) in different contexts (heterozygous or homozygous; SNVs or Indels) for each method and found homogeneous results, ranging from 0.472 to 0.478 for the heterozygous variants (SNVs and Indels) and from 0.991 to 0.993 for the homozygous ones. In addition, the variability of the distribution was very low (Supplementary Table S3). Unsurprisingly, when we estimated the allele balance for the variants detected by only one or two methods, the mean decreased to 0.340–0.376 for the heterozygotes, with a substantially higher standard deviation.

Finally, the transition to transversion (Ts/Tv) ratio within the common regions was computed and results were very similar, ranging from 2.80 to 2.83 (Fig. 1J, Supplementary Table S3). The quality scores associated to the variant calls (QUAL score and RMS Mapping Quality, Fig. 1K,L, Supplementary Table S3) were in concordance with the previously assessed parameters, with NimbleGen presenting the highest scores, followed by SureSelect^QXT^ and NRCCE, respectively.

### Pseudogenes

*STRC*, responsible for recessive NSHL, was one of the genes included in our panel. This gene is located in a large segmental duplicated region. The duplicated region contains several pseudogenes, including *pSTRC*, *STRC* pseudogene, which contains a stop codon in exon 20. *STRC* and *pSTRC* show a high level of homology, with 98.9% of genomic and 99.6% of coding sequence identity^24^. In our study, the mean coverage for *STRC* was high, ranging from 194X to 342X, and all the regions were covered >100 X. However, when we observed the alignment in the IGV genome browser, we realised that the mapping quality in *STRC* was very weak as shown on Supplementary Fig. S3.

## Discussion

The three libraries preparation methods assessed in this work present substantial differences in their specificities. Indeed, the DNA input is considerably higher in NimbleGen (500 ng versus 50 ng), which in addition uses mechanical DNA fragmentation. If the NimbleGen protocol, considered as the reference based on previous studies^13^,^14^,^15^ and our own experience^25^, is a time consuming but cost efficient method, it results in high quality libraries when the protocol is properly mastered. The two other methods evaluated here are recent and faster. They require less DNA input, use enzymatic fragmentation and do not require an additional instrument. We therefore decided to evaluate their efficiency in a clinical context.

Raw data of high quality were obtained from each library protocol, with about 5 Gb of output for each run, which represents quite a high yield for an Illumina MiSeq platform with v2 reagents (Supplementary Table S3).

We observed a high difference in the number of duplicate clusters between the three methods. The mean DOC, once duplicate reads removed, was significantly lower with SureSelect^QXT^ and even lower for NRCCE, (Fig. 1E-F, Supplementary Table S3). For the latter, nearly half of the data generated became therefore useless for variant calling. It is recognized that low DNA input and enzymatic genomic fragmentation can increase duplicates proportions^26^ as transposons systems have a marked insertion bias^27^. Moreover, we observed a wide distribution of duplicate reads, indicating that they were a direct consequence of PCR amplifications and not region-dependent (Supplementary Fig. S1).

The reduced time of contact of RNA probes with fragmented genomic DNA (hybridization) with the SureSelect^QXT^ protocol requires, to be efficient, longer probes than for other protocols (120 versus 80 bp, Supplementary Table S1). If we observed indeed a longer size of sequenced DNA fragments with the Agilent protocol, we also noticed a higher dispersion of this size. This higher dispersion (p-values < 0.0001 both compared with NRCCE and NimbleGen) could be a marker of a less efficient fragmentation method in terms of uniformity. Indeed, when comparing NRCCE and NimbleGen, a similar mean fragment size was observed but unsurprisingly the dispersion was higher with NRCCE (p < 0.0001).

Proper comparison of library preparation methods for DNA sequencing requires indicators computed to normalize the results. Our concerns in this study relied first on a slight difference in size between the three libraries, because NimbleGen and SureSelect^QXT^ designs were composed of 781 kb versus 695 for NRCCE design. The Enrichment Factor^18^ compares the ability of each protocol to enrich in regions of interest and therefore removes biases due to the different library sizes. NRCCE gave the best results, with an EF about 20% higher than with SureSelect^QXT^. This was correlated with a higher raw on target percentage of reads and bases (Fig. 1C, Supplementary Table S3). This figure demonstrates that the NRCCE protocol, while built with far less probes (about 13,000 versus 25,000 for SureSelect^QXT^ for example) had the ability to correctly select DNA sequences of interest. The robust strategy of NimbleGen based on a high number of probes (each nucleotide of the design being covered with a mean of 7 probes) combined with more than 60 hours of hybridization gave results similar to NRCCE.

A second concern, linked to duplicates, was the need for an indicator independent of the mean DOC to assess the uniformity of coverage. Our requirements in diagnostic context is to get a sequencing as even as possible, which is essential for experiment efficiency. Mokry *et al.*^23^ described the evenness score (ES), which is independent of the sequencing depth. It represents the distribution of base coverage among the targeted regions relative to the mean coverage. A theoretical score of 100% indicates a completely uniform coverage. All results obtained during the course of this study were above 80%. This percentage reflects a high uniformity of these experiments but cannot be compared with others, as ES remains dependent on the targeted regions. However, some previous studies focusing on custom capture or exome context have also shown that NimbleGen libraries resulted in the highest uniformity^10^,^13^,^17^.

Coverage was assessed at 20X, which is a commonly accepted threshold for proper variant calling^28^ and at 50X, which represents a more comfortable depth in particular for indel detection. All three methods gave high percentage of regions covered at these given depth, however the NimbleGen protocol led to a sequencing in which more than 80% of targeted regions were covered with a mean of at least 280 reliable reads (Fig. 2). This results from the high mean DOC and the high ES. This could be of importance as this may allow a higher multiplexing of the samples.

We also assessed the behaviour of each method in different GC content contexts. It has for long been demonstrated that regions of low (<30%) or high (>60%) GC content usually result in poor DOC^11^,^15^. The low coverage of these regions may be due to a bias in PCR amplification (regions with a neutral GC content are more efficiently amplified) or during the hybridization step (these regions may have a reduced capture effectiveness)^26^,^27^,^28^,^29^. We clearly showed a smaller dispersion observed with the NimbleGen library, while NRCCE is more efficient than the others in low GC content context, and SureSelect^QXT^ in high. This is quite unexpected and could be explained by the fact that SureSelect^QXT^ generates longer fragments. These results are slightly different from those obtained by Clark *et al.*^15^, who reported an efficient behaviour of Agilent SureSelect in exome sequencing context in GC-poor regions. Differences may be linked to the method, which differs from Agilent SureSelect to the faster method QXT. It has been also demonstrated that the polymerase used for amplification can be critical. Quail *et al.*^30^ compared the efficiency of several polymerases and showed that the Kapa HiFi system (Kapa Biosystems) was the most performing one, as it generates less biases.

More than 87% (677/770) of all reported variants, in mean per sample, were common between the three methods. It is worth noting that this figure includes filtered variants (e.g. variants of low proportion of supporting reads, or with a low amount of reads in the region…). The remaining 13% were identified by only 1 or 2 methods. This was explained by the lack of coverage and the variant frequency disequilibrium due to the presence of repetitive elements, N-repetitions or high GC content in the direct vicinity of the site. Moreover, 50% of these variants were located in the same region, especially in exon 5 of the gene *DSPP* (hg19:chr4:88534937–88538025, Supplementary Fig. S4), rich in repetitive sequences.

Absence of a 6 bp deletion in the NRCCE variant dataset (representing less than 20% of the reads) is either due to a software default or to a particular filtering policy. To our knowledge, the variant should have been marked by MSR as ‘LowVariantFreq’ (see “MiSeq reporter Software Guide”, Illumina), but should have still been present in the final VCF file. The identification was possible with another pipeline based on the most recent version of GATK, which performs indels realignment. Some studies have shown that long probes and baits could improve the detection of small indels^11^,^15^ and indeed this variant could be identified with SureSelect^QXT^ with an allele balance close to 35% (34.7%, 98/282 reads). On the other hand, the probe size used with NimbleGen is similar to the one used in NRCCE (75 versus 80, respectively), and the variant call was also supported by >30% of reads (116/359, 32.3%), but NimbleGen benefits from a longer hybridization time.

Ts/Tv ratio is a measure of the specificity of SNP calls; low ratios are associated with a higher probability of false positive calls. Previous studies^31^,^32^,^33^ showed ratios of about 2 for whole genome sequencing and around 2.8–3 for high quality exomes datasets. We evaluated Ts/Tv ratio in our datasets and the three methods obtained similar values of 2.80–2.83, comparable to those observed from exome data.

Presence of pseudogenes is a dreadful issue in NGS because their contamination can interfere with the detection of variants in the genuine gene and generate false positive and negative variants. Seventy percent of the *PDK1* sequence, a gene involved in autosomal dominant polycystic kidney disease, is duplicated, at least three times, across the genome with more than 95% homology. In 2013, Qi *et al.*^34^ analysed *PDK1* and the percentage of confirmed variants in the duplicated regions was 26.6 and 50% for WES and targeted capture, respectively.

In our gene panel we had to deal with *STRC* and its pseudogene *pSTRC*, for which only 56 single positions diverge among exons 16 to 28^35^,^36^. Alignments of *STRC* showed regions of poor mapping quality and we were not able to discard the inclusion of pseudogene reads. To avoid co-capture of homologous sequences, PCR based methods are more reliable as specific primers can be designed in regions with variant divergence^37^. Moreover, it is essential to be aware of the existence of pseudogenes when consulting general databases, as data resulting from WES or WGS could be contaminated with pseudogene variants.

In the present study, we have analysed several sequencing parameters to compare the performance of three enrichment methods. Significant differences are reported (i.e. evenness or on target percentage) but the three capture methods generated suitable datasets and are advisable to analyse a panel of standard genes. Although NimbleGen showed a global better performance, all methods have their pros and cons depending on the objectives of the project. If turn around time and/or DNA quantity are the main criteria, SureSelect^QXT^ or NRCCE have to be considered. However, in our own experience, NimbleGen still offers the best choice in terms of costs and overall quality.

## Methods

### Patients

Twenty-four patients suspected of USH (12 patients) or NSHL (12 patients) were selected for the study. DNA was extracted from the peripheral blood samples using standard techniques. This study was approved by the local Ethics Committee and was conducted in accordance with the Declaration of Helsinki. Informed consent for genetic testing was obtained, after explanation of the nature of the study and its possible implications to patients and families.

The 24 patients were analysed with the three different capture methods over six different runs (two independent runs of 12 patients for each method). Prior to this study, several patients had one or several genes analysed by Sanger, accumulating 113 variants suitable for NGS data validation.

### Design

Designs were performed using specific online tools provided by each company, except for NimbleGen, for which the design was performed together with the bioinformatics support (although an online tool is also available). The first (NRCCE) design included 112 genes (involved in USH, ARRP or NSHL). The target regions for each gene were defined and consisted in all the exons identified in NCBI RefSeq+/− 50 intronic flanking bp. An updated design was used for NimbleGen and SureSelect^QXT^ that included nine additional genes and the removal of one. By default, NRCCE positioned probes in all regions, including interspersed repeats and low complexity regions. For NimbleGen and SureSelect^QXT^ an intermediate stringency was selected for repetitive regions. The main characteristics of each design are showed in Supplementary Table S1.

### Enrichment methods

The six libraries (two per protocol) were performed following the instructions from the different manufacturers “Nextera Rapid Capture Enrichment Guide, August 2013”, “SureSelect^QXT^ Target Enrichment for Illumina Multiplexed Sequencing, Version C0, January 2015” and “NimbleGen SeqCap EZ Library SR, Version 4.2, December 2013”. Details and main differences of each protocols are shown in Supplementary Table S2.

All experiments were performed following the manufacturer’s protocols, with the exception of the quantity of DNA input for NimbleGen (500 ng versus 1 μg specified in the protocol).

#### NRCCE (Illumina, San Diego, CA, USA)

50 ng/μl of DNA were fragmented by enzymatic digestion and the adaptors were ligated (*tagmentation*) simultaneously. After *tagmentation*, a first amplification was performed using primers containing a specific index for each patient DNA. In the next step, 12 DNAs were pooled in the same capture reaction. Two consecutive hybridizations were then performed, the first one during 2h and a second one overnight. Library was captured using streptavidin-conjugated magnetic beads and a second PCR amplification was performed.

#### NimbleGen (Roche, Madison, WI, USA)

Genomic DNA (500 ng) was sheared by mechanical fragmentation (Bioruptor^®^ Pico, Diagenode, Liège, Belgium). Fragments were end-repaired, A-tailed, and ligated to the specific adapters. The libraries were amplified with primers specific for the adaptors and were then hybridized to the designed biotinylated probes for 66–72 h at 47 °C. Up to four DNA patients libraries were pooled in the same reaction. The biotinylated probes-DNA hybrids were recovered and purified with streptavidin-conjugated magnetic beads and a second PCR amplification was performed.

#### *SureSelect* ^*QXT*^ *(Agilent Technologies, Santa Clara, CA, USA)*

50 ng of genomic DNA was fragmented and adaptors were added in a single enzymatic step. The adaptor-tagged DNA library was purified and amplified. Next, 750 ng of each library was hybridized using SureSelect^QXT^ capture library for 90 minutes. The resulting libraries were recovered using streptavidin magnetics beads, and a post-capture PCR amplification was carried out.

### Sequencing

Final libraries were quantified with a Qubit High Sensitivity kit (Invitrogen, Carlsbad, CA) and the quality of the library was assessed on a Bioanalyzer High Sensitivity DNA chip (Agilent). Twelve patients samples were included per run.

Sequencing of the libraries was carried out on a MiSeq instrument (Illumina) according to the manufacturer’s protocol (“Preparing libraries for sequencing on the MiSeq”, October 2013). A final library concentration ranging from 8 to 10 pM was used to carry out cluster generation and sequencing on a Standard Flow Cell and MiSeq Reagent Kit v2 300 cycles (2 × 150 cycles).

### Data analysis

We used the same analysis pipeline for the 3 methods and all the runs. The sequences were aligned and mapped against human genome version 19 by MiSeq Reporter (MSR) v2.2.31 (Illumina). This software suite was chosen as it represents the analysis pipeline that would be used in molecular diagnostic laboratories that do not have access to a bioinformatics facility support.

MSR performs demultiplexing of the samples, generation of FASTQ files, and alignment against the reference with BWA software^38^. Resulting primary BAM files (alignment files) are then treated with Picard (http://broadinstitute.github.io/picard) to mark duplicates, GATK^39^ to perform realignment around indels and SAMtools^40^ for merging, sorting and indexing mature BAM files. These files are then processed with GATK’s Unified Genotyper for variant calling to generate VCF files (variant files), which are further filtered and annotated against dbSNP (version 137 in this study).

Specifications of SAM/BAM and related high-throughput sequencing file formats (including VCF) can be found at https://github.com/samtools/hts-specs.

However, this version of MSR has some drawbacks, especially in the identification of indels with an allele balance of 20–25%, as observed during the course of this study. Therefore, for a particular sample, we reanalysed NRCCE data with a custom pipeline in development in our lab based on GATK best practice for DNA sequencing (https://www.broadinstitute.org/gatk/guide/best-practices? bpm=DNAseq).

We assessed the accuracy of the different platforms calculating several quality metrics based on published guidelines^20^,^21^,^22^. For most statistical analyses, depth of coverage (DOC) computations and variant analyses, only the common regions between the 3 designs were taken into account (1,984 regions covering 678 kb). MSR and Illumina Sequencing Analysis Viewer (SAV) were used to generate some of the metrics examined in this study, as well as SAMtools to specifically estimate the DOC excluding PCR duplicates. Custom Perl scripts (https://www.perl.org/) were written to extract raw data concerning specific common regions, compare VCF files and compute Evenness Score (ES) as described in Mokry *et al.*^23^ and Enrichment Factor (EF) as described in Ware *et al.*^18^. EF was calculated to compare the libraries as it describes enrichment regardless of the size of the library, and ES because it expresses the uniformity of the coverage independently of the sequencing depth.

Normalised DOC expressing dispersion has been computed using equation (1):


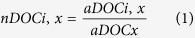


with *nDOCi, x* being normalized DOC for region i and method x; *aDOCi, x*, average DOC for region i and method x, *aDOCx*, global average DOC for method x (all regions).

The R software^41^ was used to perform statistical tests. Data for each sample were considered individually, therefore 24 values per method were compared using non-parametric unilateral Wilcoxon signed rank tests (α = 0.025). The Integrative Genomic Viewer (IGV^42^) was also used for data visual inspection.

## Additional Information

**How to cite this article**: García-García, G. *et al.* Assessment of the latest NGS enrichment capture methods in clinical context. *Sci. Rep.* **6**, 20948; doi: 10.1038/srep20948 (2016).

## Supplementary Material

Supplementary Information
