# Supplementary material for: Assessment of the latest NGS enrichment capture methods in clinical context
Source: Sci Rep. 2016 Feb 11;6:20948. doi: 10.1038/srep20948 (PMC4750071; doi:10.1038/srep20948)
Supplement: Supplementary Information [file srep20948-s1.pdf]

Assessment of the latest NGS enrichment capture methods in clinical context

Gema García-García, David Baux, Valérie Faugère, Mélody Moclyn, Michel Koenig, Mireille Claustres, Anne-Françoise Roux

Supplementary Information

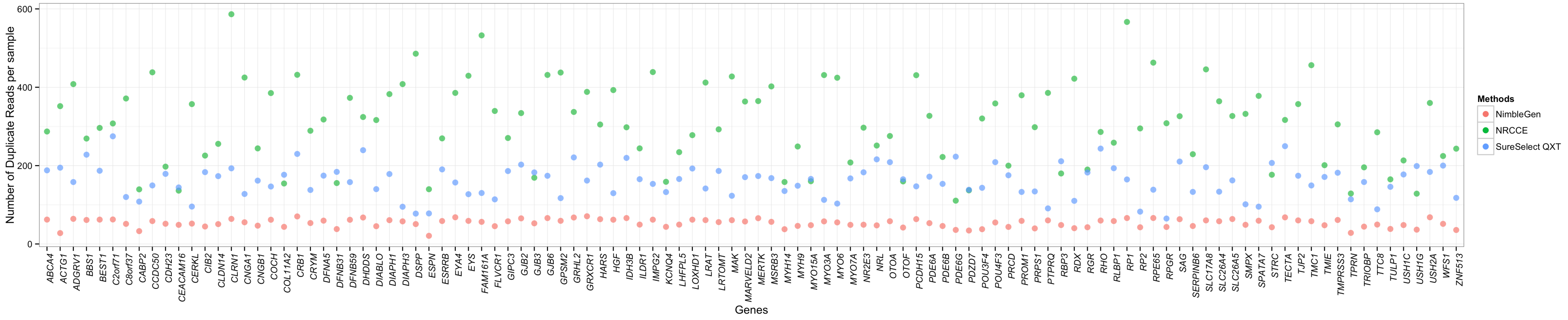

Supplementary Figure S1: Distribution of duplicate reads along the studied genes.

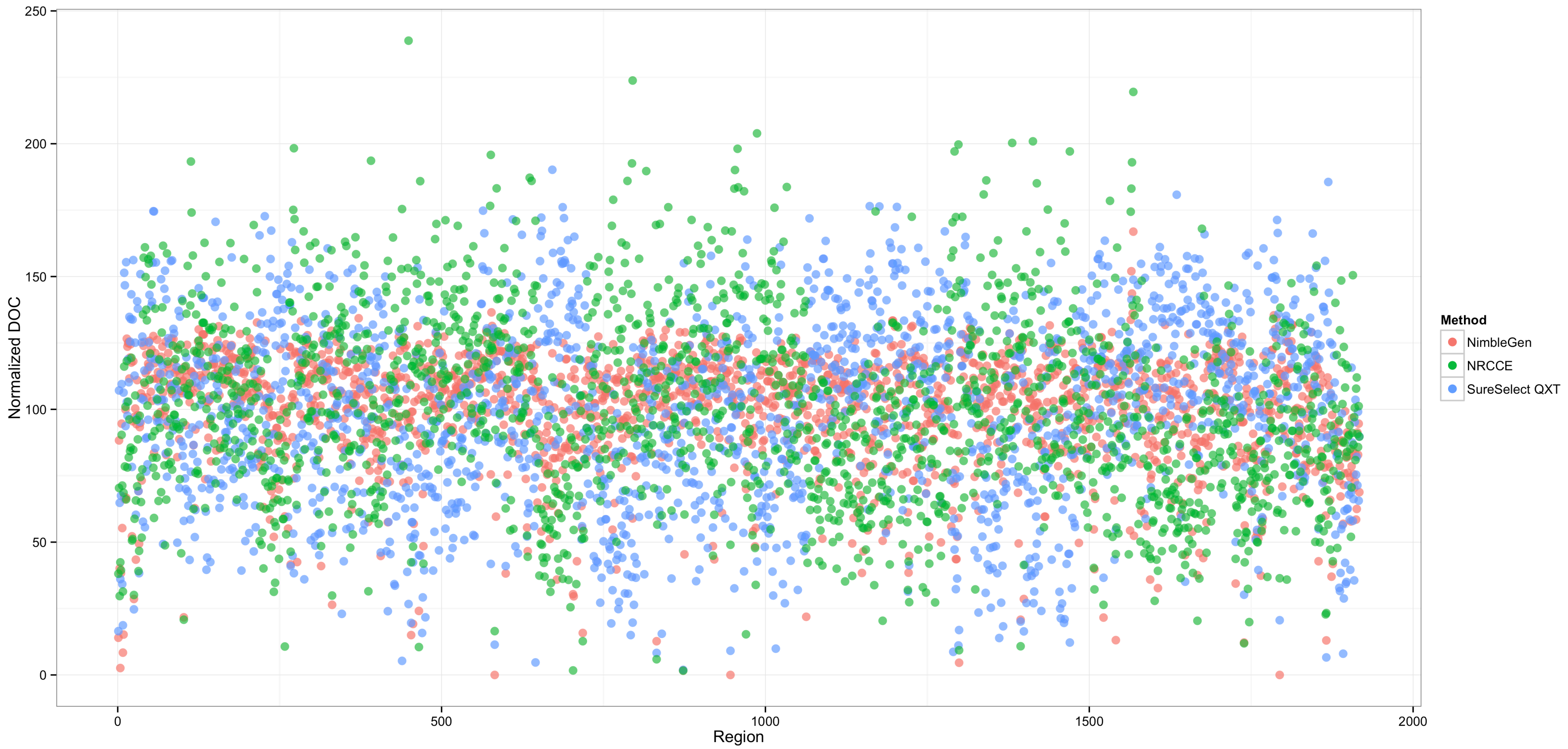

Supplementary Figure S2: Mean normalised DOC of the three methods for the 1,984 common regions.

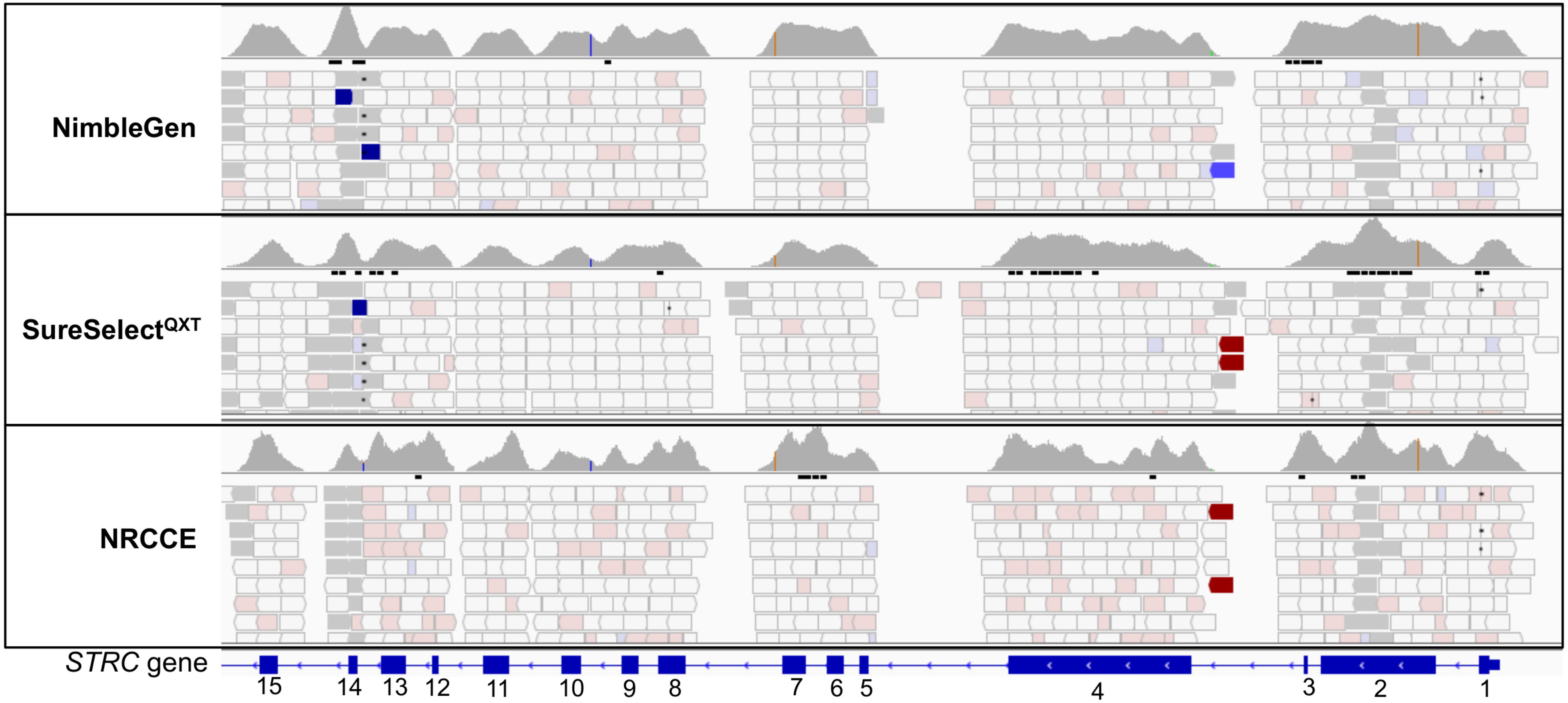

Supplementary Figure S3: IGV view of *STRC* gene sequence for the three methods. Unfilled reads represents reads of poor genomic specificity.

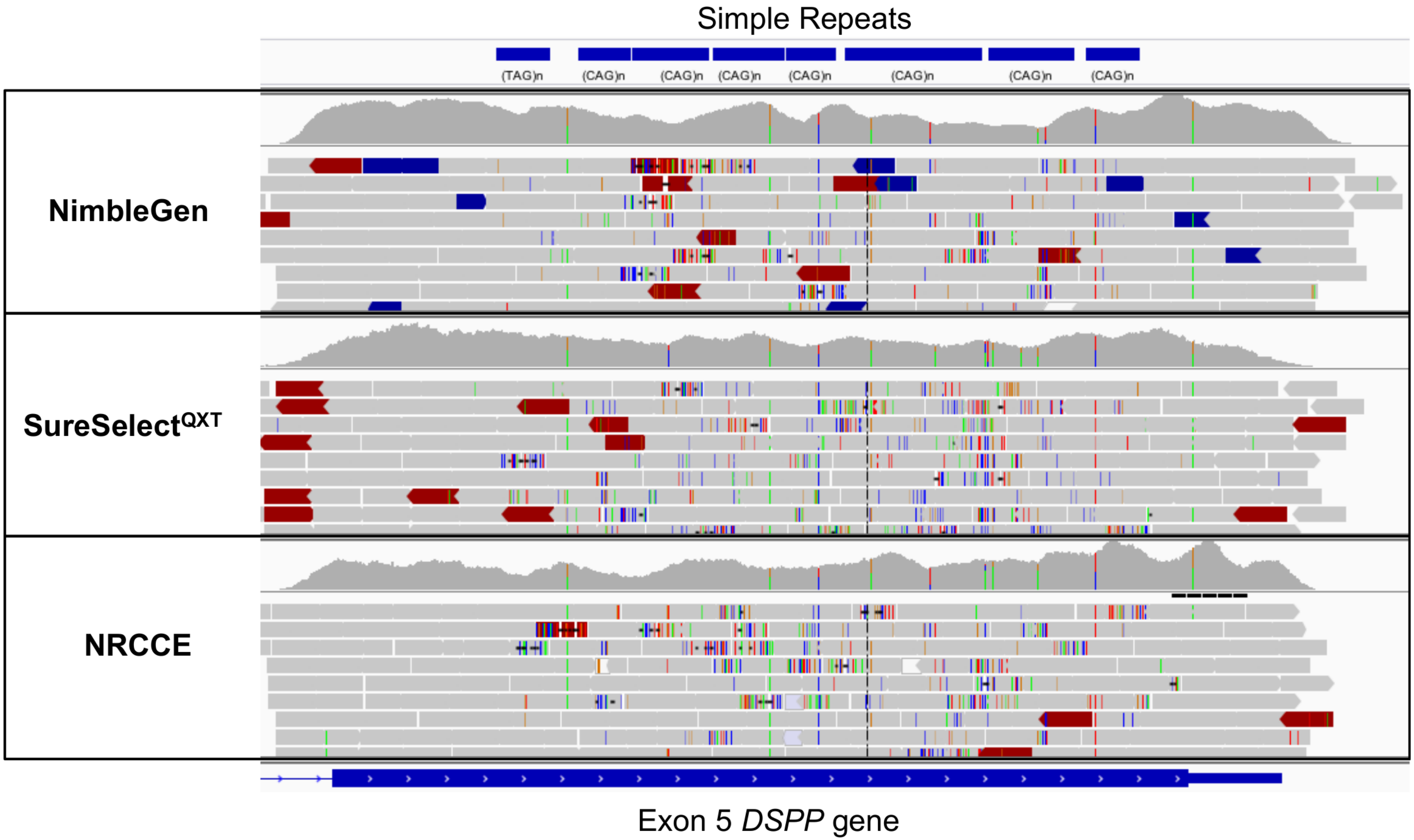

Supplementary Figure S4: IGV view of *DSPP* sequence of exon 5 for one DNA. The low complexity of the genomic sequence results in poor mapping. Each vertical bar or horizontal mark inside the reads represents alignment mismatches.

	SureSelect QXT	NRCCE	Nimblegen
**Company**	Agilent	Illumina	Roche
**Number of genes**	121	112	121
**Target size (kb)**	781	695	781
**Online tool**	SureDesign	DesignStudio	NimbleDesign
**Bioinformatics support offered**	Yes	No	Yes
**Probes size**	120	80	75.75 (+/-4.62)
**Number of probes**	30961	12966	NA
**Probes density**	Overlapping (96bp)	Overlapping (20bp)	Overlapping (NA)
**Mean probes per base**	5	1	7
**Design stringency**	Intermediate	Relaxed	Intermediate
**Percentage of target covered**	99.7	100	99.1

Supplementary Table S1: Main characteristics of the three probe designs

NA: not available

	SureSelect^{QXT}	NRCCE	NimbleGen
**Initial DNA amount (ng)**	50	50	500
**Fragmentation type**	Enzymatic	Enzymatic	Mechanic
**Number of PCR cycles pre- capture**	8	10	7
**Pool of patients before hybridization**	No	Yes (12 patients / capture)	Yes (4 patients / capture)
**DNA Amount of each patient for hybridization (ng)**	750	500	250
**Hybridization cycles (time)**	One (90 min)	Two (2h + o/n)	One (66-72h)
**Number of PCR cycles post-capture**	14	12	14
**Addition of Index**	After capture	Before capture	Before capture

Supplementary Table S2: protocol characteristics of the 3 library preparation methods

o/n: overnight

A: Agilent; I: Illumina; N: NimbleGen

	SureSelect QXT (A)	NRCCE (I)	Nimblegen (N)	Wilcoxon signed-sum test unilateral hypothesis					
**Library parameters (Means on 2 independant runs- per sample)**				H_0	p-value	H_0	p-value	H_0	p-value
Median Insert Size	224.83	187.50	187.63	I<N	0.9173	I<A	9.64E-06	N<A	9.61E-06
Insert Size SD	76.58	46.75	35.96	I>N	1.64E-04	I<A	9.66E-06	N<A	9.65E-06
% of duplicates reads	18.59	41.24	7.77	I>N	5.96E-08	I>A	5.96E-08	N<A	9.69E-06
**Run parameters (Means on 2 independant runs - per run)**									
Clusters density (K/mm2)	1090	963	1070						
Bases sequenced (Gb)	5.10	4.80	5.20						
% Q30	89.05	90.15	91.60						
# clusters raw	20197145	18131316	20144285						
% of Clusters passing filter	90.08	91.95	91.53						
% of duplicates clusters	12.88	19.63	3.64						
% of unindexed clusters passing filter	2.77	3.10	2.85						
% of unaligned clusters passing filter	0.78	0.92	1.72						
**Run parameters (Means on 2 independant runs - per sample)**									
# aligned reads	2885743	2614487	2866327	I<N	0.06776	I<A	0.09375	N<A	0.78880
% of ontarget reads	75.09	85.78	86.88	I<N	0.01913	I>A	5.96E-08	N>A	9.69E-06
# aligned bases	415890897	347747806	405126563	I<N	0.01056	I<A	0.00894	N<A	0.70810
% of on target bases	66.30	75.26	73.95	I>N	0.01548	I>A	5.96E-08	N>A	5.96E-08
*% nts > 20X**	99.37	99.04	99.32	I<N	0.01070	I<A	0.00318	N<A	0.19720
*% nts > 50X**	97.36	95.89	98.58	I<N	0.00012	I<A	0.01699	N>A	0.00037
*Mean DOC (+duplicates)**	350	371	374	I<N	0.31150	I>A	0.34200	N>A	0.06401
*Mean DOC (-duplicates)**	263	217	349	I<N	4.53E-05	I<A	0.01664	N>A	0.00117
**Variant parameters (Means per sample - AB for variants with filter 'PASS' only)**									
*# SNVs**	677	663	674	I<N	0.00033	I<A	9.61E-06	N<A	0.02948
*Mean AB (SNVs/het)**	0.47	0.48	0.47						
*SD AB (SNVs/het)**	0.07	0.08	0.07						
*Mean AB (SNVs/hom)**	1.00	1.00	1.00						
*SD AB (SNVs/hom)**	0.01	0.01	0.01						
*# Indels**	56	50	56	I<N	2.90E-04	I<A	2.07E-05	N>A	0.99880
*Mean AB (Indels/het)**	0.45	0.45	0.46						
*SD AB (Indels/het)**	0.10	0.10	0.10						
*Mean AB (Indels/hom)**	0.96	0.96	0.96						
*SD AB (Indels/hom)**	0.05	0.05	0.04						
*Ts/Tv ratio**	2.80	2.82	2.81	I>N	0.004248	I>A	0.16500	N>A	0.97610
*QUAL score**	4485	3167	5165	I<N	4.17E-06	I<A	0.00033	N>A	0.01243
*RMSMapping quality**	35.68	33.60	37.75	I<N	0.001572	I<A	8.35E-07	N>A	0.17240
*RMSMapping quality SD**	1.40	1.27	2.45						
*Evenness(%)**	82.45	81.72	88.42	I<N	9.70E-06	I<A	0.00972	N>A	5.96E-08
*Enrichment Factor**	3203	4048	3705	I>N	9.70E-06	I>A	9.70E-06	N>A	9.70E-06

Supplementary Table S3: Parameters analysed and computed in this study with statistical p-values when relevant

SD: Standard deviation

AB: Allele Balance

RMS: Root Mean Square

SNV: Single Nucleotide Variation

**these parameters have been computed considering common regions between the 3 designs only (1984 regions)*

Allele Balances have not been statistically tested as values are substantially equal for each protocol

	Family ID	Gene	#SNV	#indels	indel size	NRCCE	NimbleGen	SureSelectQXT	Validation method
**Run 1**	**S110**	*GJB2*†	0	1	1	1/1	1/1	1/1	Sanger sequencing
	**S661-1**	*MYO6*†	1	0	NA	1/1	1/1	1/1	Sanger sequencing
	**S661-2**	*MYO6*†	1	0	NA	1/1	1/1	1/1	Sanger sequencing
	**U1120**	*MYO7A**	21	1	22	22/22	22/22	22/22	Sanger sequencing
		*CDH23**	25	0	NA	25/25	25/25	25/25	Sanger sequencing
		*CIB2**	1	0	NA	1/1	1/1	1/1	Sanger sequencing
	**U1277**	*GJB2**	0	0	NA	0/0	0/0	0/0	Sanger sequencing
		MYO15A†	1	1	1	2/2	2/2	2/2	Sanger sequencing
	**S1324**	NA							
	**U1347**	*GJB2*†	0	0	NA	0/0	0/0	0/0	Sanger sequencing
		PCDH15†	1	0	NA	1/1	1/1	1/1	Sanger sequencing
	**U110**	NA							
	**U1389**	*USH2A**	14	1	1	15/15	15/15	15/15	Sanger sequencing
		GPR98†	2	0	NA	2/2	2/2	2/2	Sanger sequencing
	**U1403**	NA							
	**U1404**	*MYO7A**	11	1	2	11/12	12/12	12/12	Sanger sequencing
		*GJB2**	0	0	NA	0/0	0/0	0/0	Sanger sequencing
	**S1188**	*CDH23**	23	0	NA	23/23‡	23/23	23/23	Sanger sequencing
**Run 2**	**S370**	*GJB2**	0	0	NA	0/0	0/0	0/0	Sanger sequencing
		LHFPL5†	1	0	NA	1/1	1/1	1/1	Sanger sequencing
	**S432**	*GJB2**	0	0	NA	0/0	0/0	0/0	Sanger sequencing
	**S634**	*GJB2**	1	0	NA	1/1	1/1	1/1	Sanger sequencing
		USH1C†	1	0	NA	1/1	1/1	1/1	Sanger sequencing
		USH2A†	1	0	NA	1/1	1/1	1/1	Sanger sequencing
	**S715**	*GJB2**	0	0	NA	0/0	0/0	0/0	Sanger sequencing
	**S725**	*GJB2**	0	0	NA	0/0	0/0	0/0	Sanger sequencing
		MYH9†	1	0	NA	1/1	1/1	1/1	Sanger sequencing
	**S731**	*GJB2**	1	0	NA	1/1	1/1	1/1	Sanger sequencing
	**U1396**	*CLRN1**	0	0	NA	0/0	0/0	0/0	Sanger sequencing
		GJB2†	0	1	1	1/1	1/1	1/1	Sanger sequencing
	**U1410**	*MYO7A**	15	0	NA	15/15§	15/15	15/15	Sanger sequencing
		USH2A†	1	1	1	2/2	2/2	2/2	Sanger sequencing
	**U1415-1**	*GPR98*†	2	0	NA	2/2	2/2	2/2	Sanger sequencing
	**U1415-2**	NA							
	**U1420**	NA							
	**U1426**	*USH2A*†	0	1	[illegible]p (1 exon del	1/1	1/1	1/1	MLPA
		Total	125	8		132/133	133/133	133/133	

Supplementary Table S4: List of patients analysed

*Genes previously analysed by Sanger sequencing

†Pathogenic variants confirmed by sanger sequencing or MLPA

‡1 variant was filtered as LowDepth (7 reads)

§For this genotype NRCCE alone reported a variant chr11:g.76908662T>C with borderline allelic balance (21%, then not filtered out, the limit being 20%) - variant not confirmed by Sanger sequencing
